# Emerging**** Roles of Exosomes in T1DM

**DOI:** 10.3389/fimmu.2020.593348

**Published:** 2020-11-26

**Authors:** Haipeng Pang, Shuoming Luo, Yang Xiao, Ying Xia, Xia Li, Gan Huang, Zhiguo Xie, Zhiguang Zhou

**Affiliations:** National Clinical Research Center for Metabolic Diseases, Key Laboratory of Diabetes Immunology (Central South University), Ministry of Education, and Department of Metabolism and Endocrinology, The Second Xiangya Hospital of Central South University, Changsha, China

**Keywords:** type 1 diabetes mellitus, exosomes, biomarkers, microRNAs, therapy

## Abstract

Type 1 diabetes mellitus (T1DM) is a complex autoimmune disorder that mainly affects children and adolescents. The elevated blood glucose level of patients with T1DM results from absolute insulin deficiency and leads to hyperglycemia and the development of life-threatening diabetic complications. Although great efforts have been made to elucidate the pathogenesis of this disease, the precise underlying mechanisms are still obscure. Emerging evidence indicates that small extracellular vesicles, namely, exosomes, take part in intercellular communication and regulate interorgan crosstalk. More importantly, many findings suggest that exosomes and their cargo are associated with the development of T1DM. Therefore, a deeper understanding of exosomes is beneficial for further elucidating the pathogenic process of T1DM. Exosomes are promising biomarkers for evaluating the risk of developingty T1DM, monitoring the disease state and predicting related complications because their number and composition can reflect the status of their parent cells. Additionally, since exosomes are natural carriers of functional proteins, RNA and DNA, they can be used as therapeutic tools to deliver these molecules and drugs. In this review, we briefly introduce the current understanding of exosomes. Next, we focus on the relationship between exosomes and T1DM from three perspectives, i.e., the pathogenic role of exosomes in T1DM, exosomes as novel biomarkers of T1DM and exosomes as therapeutic tools for T1DM.

## Introduction

Type 1 diabetes mellitus (T1DM) is an autoimmune disorder characterized by beta-cell dysfunction and death caused by autoreactive T cells, an absolute lack of insulin, and elevated blood glucose levels (1–3). Persistent hyperglycemia leads to the development of life-threatening diabetes-associated complications such as blindness, stroke, kidney diseases, and heart diseases, thus decreasing the quality of life of patients and imposing a considerable economic burden on society and individuals.

Currently, it is widely accepted that a combination of genetic and environmental factors contribute to an increased risk of T1DM (4–6). Although substantial research efforts have been made to elucidate the pathophysiology of T1DM, the exact underlying mechanisms are still largely unknown. For example, the critical initial triggering events that result in infiltration of T lymphocytes and pancreatic islet autoimmunity, which are important in early identification of T1DM and effective prevention of further islet deterioration, have not been revealed. Because the pathogenic mechanisms are obscure, most patients with T1DM rely on life-long exogenous insulin administration, which only alleviates symptoms. Today, the most commonly used biomarkers of T1DM are human leukocyte antigen (HLA) genes and islet autoantibodies. However, these biomarkers do not fully meet current needs. The ideal biomarkers should be objective indicators of disease condition that can be measured accurately and reproducibly, should identify disease stage and progression and should assess the outcome of therapies.

Exosomes, which are small vesicles carrying bioactive molecules such as DNA, RNA, and proteins, have emerged as important mediators of cellular and interorgan communication. Evidence shows that exosomes may be involved in the loss of tolerance towards islet cells and take part in islet autoimmunity (7). Therefore, a better understanding of exosomes may provide novel insight into the onset and development of T1DM. In addition, studies have indicated that the number and composition of exosomes can reflect the physical and pathological status of their cells of origin, which means that monitoring exosomes can be helpful for disease diagnosis. Therapeutically, exosomes have the potential to be exploited as novel treatment agents and drug delivery vectors. Therefore, more comprehensive knowledge of exosomes may not only help reveal the underlying pathogenic mechanisms of T1DM but also provide valuable targets for use as disease biomarkers and therapeutic tools.

## Extracellular Vesicles and Exosomes

In recent years, in addition to cytokines, chemokines and hormones, a new group of modulators called extracellular vesicles (EVs) that can regulate cell-to-cell communication have emerged (8). EVs are a group of heterogeneous lipid bilayer-enclosed structures that are secreted into the extracellular milieu by multiple types of cells. These small membrane-bound structures can be released by almost all cell types in response to endogenous and exogenous stimulation (9). According to their biogenesis, size, content and biological function, EVs can be mainly classified as exosomes (EXOs), apoptotic bodies and microvesicles (MVs) (10–12). However, given the consensus has not been reached on specific markers of EV subtypes and the fact that distinguishing the biogenesis pathway of EVs remains extremely difficult, MISEV 2018 (Minimal information for studies of extracellular vesicles 2018) recommended authors to use operational terms for EV subtypes that refer to physical characteristics of EVs, biochemical compositions, and description of conditions or cell of origin (13). But in this review, we adopt the description used by reference literature for the sake of convenience in the recital.

Exosomes, which range from 30 to 200 nm in diameter, are present in various kinds of biological fluids, such as serum, cerebral spinal fluid, saliva, urine, pleural effusion or ascites, and breast milk (14–16). They can mediate intercellular communication *via* cargo molecules. The cargo delivered by EXOs includes DNA, RNA (miRNA, tRNA, mRNA, rRNA), proteins and lipids (8, 17). Because the cargo of EXOs can reflect the status of their cells of origin, monitoring and repurposing these nanovesicles can be useful for the diagnosis and therapy of many diseases, including type 1 diabetes mellitus (T1DM). EXOs are formed through endosomal networks, and they thus bear specific markers such as tetraspanins (CD9, CD63, and CD81), heat shock proteins (HSP70), and the Rab family proteins Tsg101 and Alix (18, 19). The biogenesis of EXOs can be divided into three stages: (1) the invagination of early endosomes, which engulfs content from the cytoplasm; (2) the formation of multivesicular bodies (MVBs) *via* inward budding of the endosomal membrane; and (3) the fusion of MVBs with the plasma membrane and secretion of exosomes (20). The biogenesis of EXOs is strictly regulated by multiple factors, such as the cell type (14), contact inhibition (21, 22), cell culture (23), Ca^2+^ (24), and hypoxia (25). More importantly, many pathological states, such as cancer (26, 27), diabetes (28), and neuronal degradation (29, 30), affect the yield and content of exosomes, making it theoretically feasible to apply exosomes for the diagnosis and treatment of diseases. Upon release, these exosomes can induce biological responses of recipient cells *via* a range of processes, including protein-protein interactions on the cell surface or entry into the cytosol of recipient cells through endocytosis and fusion with the plasma membrane (28). Compared with exosomes, the biogenesis of the MVs (50–2,000 nm in size) is far less learned. In general, the formation of MVs is resulted from dynamic interplay between phospholipid redistribution and cytoskeletal protein contraction, which is distinct from the biogenesis of exosomes (31). Also, the cargo of MVs tends to be highly enriched for specific proteins which are different from exosomes. For example, a study indicated that the MVs secreted by melanoma cells are enriched for B1 intergrin receptors (32). On the other hand, transferrin receptors, which are highly detected in exosomes, are missing in MVs (33). Unlike exosomes and MVs are produced during normal cellular process, the formation of apoptotic bodies (500–4000 nm in size) is associated with programmed cell death. This process is characterized by condensation of the nuclear chromatin, membrane blebbing, and the cellular content enclosed by apoptotic bodies (34). Most apoptotic bodies will be eliminated by macrophages locally (35).

Given the emerging roles of exosomes in multiple physiological and pathogenic processes, extensive effort has been applied to further understand exosomes and improve their isolation methods. A variety of technologies, including ultracentrifugation, affinity-based capture technology, filtration, chromatography, precipitation, and microfluidics, have been developed to isolate EXOs. However, given that the size of EXOs is extremely small, isolation is very challenging, and all these techniques have their own limitations (14). Therefore, it is imperative to develop or advance new or existing methods to isolate exosomes.

## The Pathogenesis of T1DM

The pathogenesis of T1DM is associated with a complex interplay between genetic and environmental factors. However, the early triggering events of T1DM are still largely unknown. It is of particular importance to elucidate the factors that lead to beta-cell-specific T cell intolerance; these factors may include genetics, exogenous infection, endogenous superantigens, physiological stress events, and noninfectious environmental elements (36). In nonobese diabetic (NOD) mice, an excellent animal model representing human T1DM, the breakdown of tolerance to pancreatic islet self-antigens occurs spontaneously in early life. Before lymphocyte infiltration, physiological abnormalities of the islets, including vascular pathology, increased endoplasmic reticulum (ER) stress, and enhanced expression of inflammatory cytokines, are present in the pancreas in NOD mice (37, 38). These events may lead to beta-cell dysfunction and death, thus leading to the release of autoantigens and the activation of specific autoreactive T cells.

In addition, some reports show that stromal cells, rather than endocrine cells, might be critical factors inducing local inflammatory responses and subsequent islet autoimmunity. For example, peri-islet Schwann cells have been proposed as early targets involved in the initial peri-insulitis, and a specific population of T cells targeting Schwann cell antigens has been identified (39, 40). However, given that these cells do not express candidate antigens of T1DM and some lymphocyte-infiltrated islets do not undergo the peri-insulitis stage, it can be speculated that Schwann cells are not the only contributor. Additionally, islet endothelial cells (IECs) are associated with early triggering events of T1DM because they may facilitate the infiltration of autoreactive T cells into islets (41). Moreover, it has been reported that lymphatic vessel endothelial cells are also involved in islet inflammatory responses (42). It is more likely that both beta-cells and stromal cells contribute to early triggering events. Interestingly, some findings have indicated that exosomes can mediate communication between different cell types within the islets and have immunostimulatory as well as immunomodulatory properties, suggesting that they might serve as early agents inducing the initial events of T1DM (43).

## The Potential Role of Exosomes in T1DM

**Figure 1:**
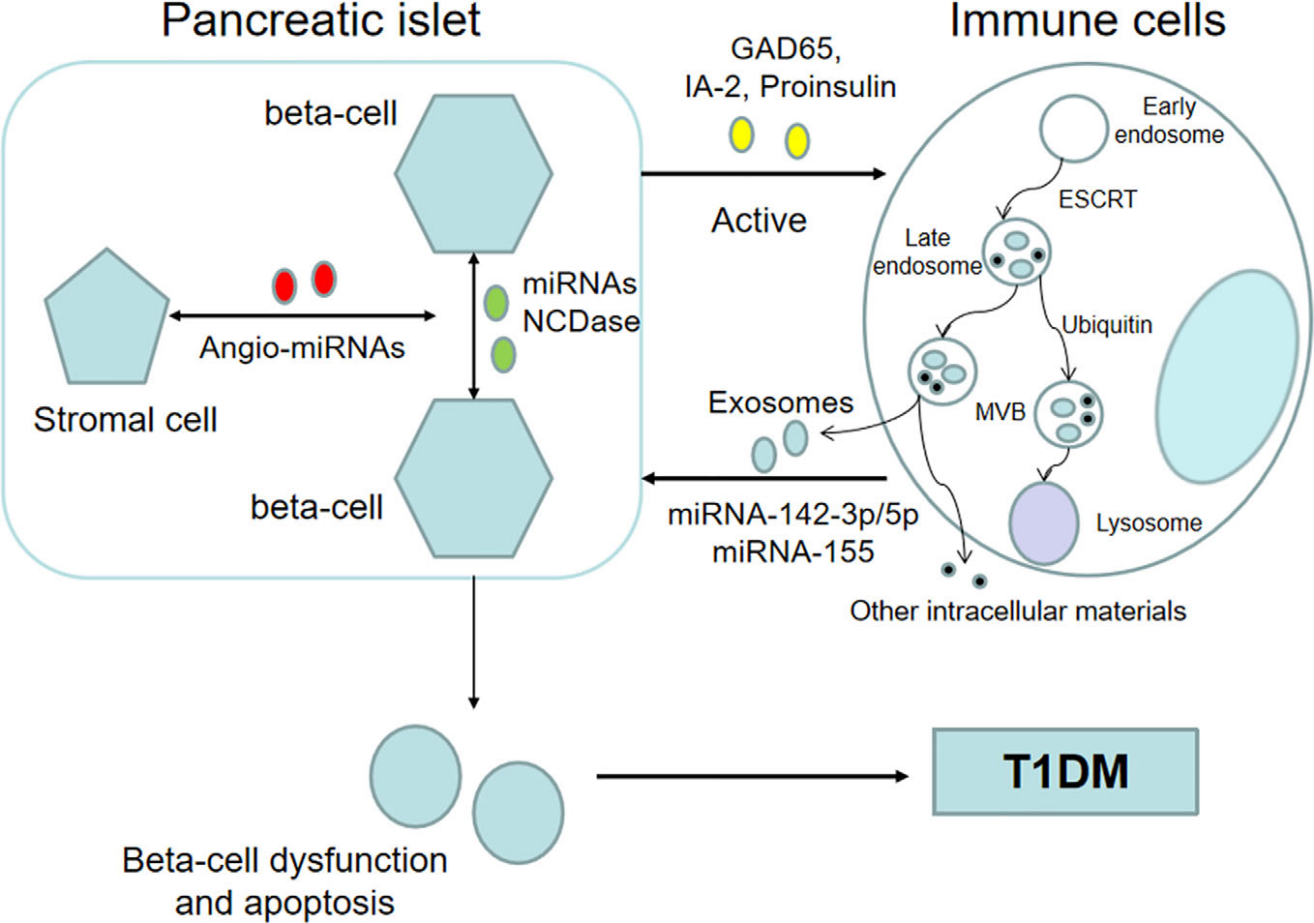
Exosomes participate in the pathological process of T1DM. Beta cell-derived exosomes that contain islet autoantigens and specific miRNAs can activate the immune system. In return, immune cell-derived exosomes can induce beta-cell dysfunction and apoptosis, eventually leading to T1DM. In addition, exosomes can deliver biological information between beta-cells, and horizontal message transfer can coordinate beta-cell activity. Additionally, some studies have shown that exosomes may serve as mediators between insulin-producing beta cells and stromal cells and are associated with the revascularization process after islet transplantation.

Emerging evidence has indicated that exosomes, which possess immunoregulatory functions, may participate in the initiation and development of autoimmune diabetes (**Figure 1**) (28, 44). On the one hand, islet-derived exosomes can activate the immune system and lead to autoimmune responses (**Table 1**) (53). At present, the exact mechanisms by which intracellular autoantigens are initially detected by the immune system and presented to autoimmune T cells have not been fully elucidated in the pathogenesis of T1DM. Intriguingly, a recent finding indicates that rat and human pancreatic islets can release exosomes containing beta-cell autoantigens that belong to intracellular membrane proteins, including glutamic acid decarboxylase 65 (GAD65), islet-associated protein 2 (IA-2) and proinsulin, and released exosomes can be taken up by dendritic cells and lead to cell activation (45). Moreover, the anchoring of GAD65 to exosome-mimetic liposomes, whose size and lipid composition is similar to islet exosomes, can enhance antigen presentation and T cell activation in individuals susceptible to T1DM (45). In addition, a previous study indicated that mouse MIN6 insulinoma cells can release exosomes that express GAD65 (50). These studies indicated that exosomes might be important agents in the development of T1DM. However, there are some problems remained to be clarified. For example, because the findings are drawn based on *in vitro* experiments, *in vivo* studies are necessary. In addition, apoptotic beta-cells can also release autoantigens which can be taken by APCs in pancreatic lymph nodes and activate autoimmune responses. So whether exosomes are primary drivers in the initiation of autoimmune responses against pancreatic beta cells or rather are secondary contributors in the development of T1DM needs further investigation. But given the secretion of exosomes is a positive process and can occur before beta-cell destruction, they are seemed to play more critical role in the initiation of autoimmune responses.

**Table 1:** Summary of findings on exosomes and T1DM.

Experimental subjects	Findings	References
Rats and humans	Exosomes released from the pancreatic islets contain beta-cell autoantigens and can activate adaptive immune responses	(45)
MIN6B1 cells	Exosomal miRNA transfer regulates the activity of beta-cells and transduces apoptotic signals	(46)
NOD mice	Exosomes released from islet-derived MSCs can trigger autoimmune responses in NOD mice	(7)
INS-1 cells	NCDase-containing exosomes released by INS-1 cells inhibit beta-cell apoptosis induced by high levels of inflammatory cytokines	(47)
MIN6 and NOD mice	Exosomes containing miR-29b released from beta-cells modulate innate and adaptive immune responses	(48)
NHI6F Tu28	Pancreatic beta-cells shed membrane-derived microvesicles	(49)
MIN6 and NOD mice	Insulinoma-released exosomes can activate autoreactive T cells in NOD mice	(50)
MIN6 and NOD mice	Insulinoma-released exosomes can activate autoreactive marginal zone-like B cells in prediabetic NOD mice	(51)
Human islets and NOD mice	Exosomal miRNAs derived from T lymphocytes promote pancreatic beta-cell death	(52)

Moreover, exosomes are closely associated with physiological islet abnormalities prior to lymphocytic infiltration, including increased ER stress in beta-cells and enhanced expression of proinflammatory cytokines. *In vitro* research indicates cytokine-induced ER stress can lead to increased exosome secretion by islet cells and subsequently increased exosomal proteins such as the chaperones calreticulin, ORP150 and Gp96, which can induce immune responses *via* enhanced phagocytosis and adjuvant capacity (45, 54, 55). The increased secretion of exosomes during ER stress may be explained by two theories. First, given their role in intercellular communication, the upregulation of exosomes may deliver ER stress conditions to neighboring cells. Second, exosomes may serve as vehicles for the disposal of unneeded cell material in response to ER stress to regain homeostasis. Another *in vitro* study indicated that exosomes containing miR-29b released from beta-cells can stimulate the secretion of IFN-α, IL-10, and IL-6 by splenocytes from NOD mice (48). Also, *in vitro* study indicates that the mouse insulinoma-derived microparticles also exert a strong adjuvant effect to induce the secretion of inflammatory cytokines, including IL-6 and TNF-α, *via* a MyD88-dependent pathway. *In vivo* experiments indicate that immunization with insulinoma-derived exosomes can cause insulitis in nonobese diabetes-resistant mouse models and that EXO-reactive Th1 cells and marginal zone-like B cells are detected in prediabetic NOD female mice (50, 51). Additionally, islet-derived mesenchymal stem cells (MSCs) can release highly immunostimulatory exosomes that can cause T cell-mediated destruction of the pancreatic islets in NOD mice (7). All these findings suggest that abnormal release of exosomes may trigger early inflammation and autoimmunity in the islets. However, whether these phenomena exist in human body and play a role in physiological process await further investigation.

On the other hand, exosomes derived from the immune system may lead to dysfunction and death of beta-cells (**Table 1**). A recent study indicated that exosomes containing specific miRNAs, including miR-142-3p/5p and miR-155, released by T cells can trigger apoptosis and chemokine gene expression in islet beta-cells of NOD mice (52). These chemokines, including Ccl2, Ccl7, and Cxcl10, are involved in the recruitment of immune cells and the promotion of beta-cell death in response to autoimmune attack. But because human T1DM has some distinct features compared to NOD mice, future studies need to clarify the adoptability in human body. Moreover, another study indicated that plasma-derived exosomes from patients with T1DM exhibit deregulated miRNAs and that these miRNAs are involved in the progression of T1DM (56). Subsequent functional analysis demonstrated that human islets coincubated with exosomes from T1DM patients showed decreased insulin output in the second phase in response to glucose stimulation. This finding suggests that exosomes and their content may serve as a new communication mediator between the immune system and insulin-producing beta-cells.

In addition, exosomes can deliver biological information between pancreatic beta-cells. One study indicated that the miRNA content of exosomes originating from beta-cells is regulated by inflammatory mediators, and incubation with these cytokine-induced exosomes leads to naïve beta-cell apoptosis (46). This finding provides a novel potential communication mode to coordinate the activity of beta-cells in addition to direct cell-to-cell contact and the release of signaling molecules with autocrine and paracrine functions (57, 58). Moreover, another study indicated that low-dose cytokines can stimulate the secretion of exosomes carrying neutral ceramidase (NCDase) from INS cells and that these NCDase-containing exosomes can inhibit apoptosis induced by proinflammatory cytokines at a high concentration. Similarly, NCDase packaged in exosomes secreted from beta-cells can ameliorate palmitate-induced apoptosis in INS-1 cells (59). Although this discovery is more strongly associated with T2DM, it also suggests that horizontal message transfer between beta-cells *via* exosomes does exist and may play an important role in the pathological process of the pancreatic islets. However, all these findings are drawn based on cell experiments, *in vivo* studies are required to elucidate whether this mechanism also exists in physiological conditions and evaluate the relative contribution in mediating lateral communication between beta cells.

Notably, given that almost all cell types can secrete exosomes, there must be other pathogenic mechanisms apart from the communication mode mentioned above in the development of T1DM. For example, studies have shown that compared with those from healthy controls, breast milk-derived exosomes from mothers with T1DM contain different levels of miRNAs, and pathway analysis indicates that these miRNAs are involved in the modulation of the infant immune system (60). However, whether this increases the risk of T1DM in infants is unknown.

In conclusion, based on current knowledge, exosomes may play a critical role in the onset and development of T1DM by delivering biological information, at least between beta-cells as well as between the pancreatic islets and the immune system. Previous studies have demonstrated that a complex network formed by exosomes may collectively contribute to the onset of T1DM, but how much of a role exosomes can play remains further research.

Besides T1DM, exosomes also play a role in other autoimmune diseases, including rheumatoid arthritis, systemic lupus erythematosus, and Sjogren’s syndrome (61). It is not surprising because exosomes can be secreted by almost all cell types and has multiple biological functions, such as intercellular as well as interorgan communication and modulation of immune responses. In fact, exosomes may have broader effects on regulating physiological and pathological processes due to their universality and versatility.

## Exosomes as Novel Biomarkers of T1DM

Before the manifestation of clinical symptoms, the underlying autoimmune changes of T1DM occur, and this symptomless period offers a great opportunity to predict and prevent disease progression (62). However, suitable biomarkers to identify and stratify the high-risk population and to evaluate the efficacy of intervention measures have not been developed, as the existing biomarkers often mark the late stage of T1DM when almost 90% of beta-cells have been lost. Currently, the combination of susceptible genes and islet autoantibodies is the most useful biomarker to predict T1DM risk (63). Previous studies have identified more than 50 candidate loci; a minority of genes (*HLA*) have large effects, but a majority of these genes have small effects (2). HLA genes confer the greatest risk for the development of T1DM, and the *HLA-DR* (DR3/4) and *HLA-DQ* (DQ8) genotypes are mostly used to predict the risk of developing islet autoimmunity (64, 65). Furthermore, combined evaluations of other risk genes with smaller effect sizes than *HLA* do remarkably improve sensitivity and specificity for the identification of high-risk individuals. However, a study indicated that 90% of individuals identified through genetic markers never displayed autoimmunity, and less than 50% of cases were identified by a combination of genetic markers (66).

The appearance of autoantibodies usually precedes the clinical manifestation of T1DM by months to years (67). The major circulating autoantibodies against beta-cell peptides and proteins include GAD65, IA-2, insulin, and zinc transporter 8 (ZnT8) (68–71). Although there is no evidence that these autoantibodies contribute to the pathogenesis of T1DM directly, it has been accepted that they are hallmarks of T1DM (65). At present, autoantibodies are used as biomarkers of T1DM in the clinic, and positivity for multiple autoantibodies is associated with a higher risk of T1DM regardless of family history (63). In fact, children positive for two or more autoantibodies almost inevitably develop diabetes. However, there are some limitations regarding the clinical application of these markers (67). For example, given that the time from seroconversion to diagnosis can span from weeks to decades, other biomarkers are needed before and after seroconversion. In addition, some patients never display these autoantibodies at diagnosis, and a subset of autoantibody-positive individuals will not develop clinical diabetes (72).

Currently, exosomes are viewed as potential biomarkers for diagnosing disorders such as tumors because the molecular cargo of exosomes can reflect the cell type and status of their releasing cells (15, 73). Furthermore, exosomes have additional advantages compared with traditional diagnostic methods, including (1) secretion in easily accessible biological fluids, such as urine and blood; (2) the ability to be preserved for a relatively long time at −80°C due to their stability; and (3) enhanced molecular stability in protease- and nuclease-controlled environments (43).

In the context of T1DM, previous studies have indicated that pathophysiological conditions in the pancreatic islets affect the composition of exosomes originating from beta-cells (**Table 2**) (46, 49). Profiling of exosomal RNAs derived from human islets with T1DM has demonstrated that RNAs are differentially expressed in cells subjected to treatment with proinflammatory cytokines compared to those without cytokine treatment, and these differentially expressed RNAs are associated with insulin secretion, necrosis, apoptosis, and calcium signalling (74). This study applying *ex vivo* stress model not only provides a comprehensive map of exosomal RNA from human pancreatic islets, but also can pin down the source of these circulating molecules. Among all the components, exosomal miRNAs are particularly attractive for developing novel biomarkers of T1DM (84). Global profiling analysis applying beta-cell lines and pancreatic islets has revealed that a subset of miRNAs is preferentially secreted in exosomes, while others are prone to be retained in cells (46, 85). Moreover, an *in vitro* study indicated that the miR-21-5p cargo inside EVs is increased in response to inflammatory cytokines and has promise as a future biomarker of T1DM (86). Subsequent research indicates that miR-21-5p from serum is increased in children with new-onset T1DM compared with healthy children, and interestingly, the total serum miR-21-5p is decreased among diabetic individuals, which proves the cargo within EVs is packed selectively (86). Future study should focus on identifying EV specific proteins that facilitate enrichment for EVs originated from beta-cell. With more practical significance, another study performed plasma-derived exosome characterization and reported a distinct miRNA signature in patients with long-duration T1DM, with seven differentially expressed miRNAs compared with healthy controls (56). However, the mean duration of diabetic participants in this study is 25.3 years, which weakens the potential diagnostic value of identified exosomal RNA. In the context of T1DM, exosomes have also been identified as biomarkers of diabetic complications, including nephropathy (78, 80, 82, 83) and retinopathy (87), and may be used for noninvasive monitoring of islet transplantation outcome (76).

**Table 2:** Summary of findings on exosomes as biomarkers of T1DM.

Experimental subjects	Findings	References
Humans	Exosomal miRNAs may serve as potential circulating biomarkers of T1DM	(74)
Humans	Analysis of plasma-derived exosome miRNAs as novel diagnostic tools for T1DM	(56)
Humans	Circulating transplant islet-specific exosomes may be a novel diagnostic tool for recurrent autoimmune T1DM after islet transplantation	(75)
Humans and mice	Transplanted islet-derived exosomal miRNAs as biomarkers for monitoring immune rejection	(76)
Humans	Urinary excretion of AQP2 and AQP5 *via* exosomes as biomarkers for T1DM nephropathy	(77)
Humans	Urinary podocyte EVs may serve as early biomarkers of glomerular injury in T1DM	(78)
Humans	High levels of exosomal cytokines and angiogenic factors in plasma may serve as biomarkers of diabetic ocular complications	(79)
Humans	Increased cystatin B and altered protease profiles in urinary EVs may serve as biomarkers of kidney damage in T1DM	(80)
Rats	Decreased urinary exosomal regucalcin may serve as a biomarker of diabetic kidney disease	(81)
Humans	Urinary exosomal miR-145 may serve as a biomarker of T1DM with diabetic nephropathy	(82)
Humans	The WT1 protein in urinary exosomes may be an early noninvasive marker of diabetic nephropathy in T1DM	(83)

## Exosomes as Therapeutic Tools for T1DM

Nowadays, most patients with T1DM rely on life-long insulin administration, which can only relieve symptoms. According to existing knowledge about T1DM, the curable strategies lie in re-establishing immune tolerance, annihilate islet-reactive lymphocytes, and supplement the depleted beta-cells. Seeing that exosomes not only play a role in immune stimulation, but also in immune tolerance, they are emerging as an alternative tool to induce and rebuild auto-tolerance. Also, some stem cell-derived exosomes have been reported to protect beta-cell from autoimmune attack, slow disease progression, and improve the survival of transplanted islets.

### The Advantages of Exosomes as Therapeutic Tool

Given that exosomes can exert biological effects on target cells, they are viewed as potential therapeutic agents (**Table 3**). Both *in vitro* and *in vivo* studies indicate that exosomes can transfer bioactive molecules between cells (101, 102). As a therapeutic delivery route for functional molecules, including RNA, DNA, and proteins, or synthetic drugs, exosomes can prevent cargo decomposition. For example, the clinical application of nucleic acids as drugs has been impeded because they are easily degraded. However, this problem can be solved by packaging RNAs and their mimics inside exosomes. A study indicated that two miRNAs, miR-106b-5p, and miR-222-3p, contribute to bone morrow transplantation (BMT)-induced beta-cell regeneration in mouse models of insulin-deficient diabetes, which may lead to the development of new therapeutic tools for diabetes (90). In addition to protecting the cargo from enzymatic degradation, the use of exosomes as therapeutic vectors has some other advantages, including (1) the ability to be isolated from patients themselves to avoid an immune rejection response; (2) a widespread distribution due to their liposolubility and ability to cross the intact blood-brain barrier; (3) the ability to be modified to target specific cell types by carrying special surface proteins or receptors; and (4) a relatively long half-life in the body (44, 103–105).

**Table 3:** Summary of findings on exosomes as a potential therapeutic strategy for T1DM.

Experimental subjects	Findings	References
Human MSCs and PBMCs	MSC-derived MVs inhibit inflammatory T cell responses in the islets *via* induction of regulatory dendritic cells in T1DM	(88)
STZ-induced mouse model of T1DM	Exosomes released by adipose tissue-derived MSCs exert immunomodulatory effects upon T cells and ameliorate clinical symptoms of T1DM	(89)
Human pancreatic islets	Islet-derived EVs are involved in beta cell-endothelium cross-talk and the neoangiogenesis process and may benefit engraftment of transplanted islets	(85)
Mouse model of insulin-deficient diabetes	Exosomal miR-106b and miR-222 derived from transplanted bone morrow promote beta-cell proliferation and ameliorate hyperglycemia	(90)
STZ-induced rat model of T1DM	Stem cell-derived exosomes may regenerate beta-cells through the Pdx-1 pathway	(91)
STZ-induced rat model of T1DM	Exosomes derived from MSCs exert therapeutic and regenerative effects upon the pancreatic islets	(92)
Rat model of diabetic nephropathy	Exosomes released by human urine-derived stem cells prevent kidney injury in rats with T1DM	(93)
STZ-induced rat model of T1DM	Adipose tissue-derived MSC exosomes improve erectile function in diabetic rats	(94)
Rat model of T1DM	Exosomal miR-145 released by bone morrow stromal cells exerts neurorestorative effects in diabetic rats with stroke	(95)
STZ-induced diabetic rat model	Exosomes released by human endothelial progenitor cells promote cutaneous wound healing in diabetes	(96)
NOD *scid* gamma mouse model	MSC-derived exosomes improve islet transplantation by enhancing islet function and inhibiting immune rejection	(97)
STZ-induced diabetic mouse model	Exosomes released by bone morrow MSCs improve diabetes-induced cognitive impairment	(98)
Transgenic mouse model	Hsp20-engineered exosomes may be a potential therapeutic agent for diabetic cardiomyopathy	(99)
C57BL/6J mouse model	Exosomal miRNA let7c derived from MSCs attenuates renal fibrosis in diabetes	(100)
Rat model of T1DM	Exosomes derived from human urine-derived stem cells prevent T1DM kidney complications	(93)

### Stem Cell-Derived Exosomes and T1DM

Mesenchymal stem cells (MSCs), which can be also defined as multipotent stromal cells, possess self-renewal ability and can differentiate into other tissues. MSCs are capable to remodel the injured and inflammatory tissues and maintain homeostasis of microenvironment by directly differentiating into required cell types or secreting bioactive and soluble factors. In addition, some evidences indicate MSCs can suppress excessive immune response, such as activation of T cells and B cells, *via* their paracrine ability (106, 107). These immune regulatory characteristic of MSCs has been studied for the treatment of autoimmune disorders, such as T1DM, multiple sclerosis and inflammatory bowel disease (108). Though MSC therapy has great therapeutic potential for multiple diseases, it still has several critical limitations, such as high cost, low reproducibility, and safety issues. Inspiringly, EVs, including exosomes, seem to mirror biophysical characteristics of parent cells and convey the cell functions. Some studies have indicated that the protective paracrine effects of MSCs are at least partially mediated by EVs, that is, EVs have homologous anti-inflammatory and regenerative effects as MSCs (109).

To date, accumulated research suggests that stem cell-derived exosomes possess congenital therapeutic potential and might protect pancreatic beta-cells from autoimmune assault, thus ameliorating disease progression (105, 110). It has been reported that exosomes isolated from menstrual blood-derived MSCs enhance beta-cell regeneration and insulin secretion through the pancreatic and duodenal homeobox 1 pathway in rat models of T1DM (91). However, there is no significant impact on non-fasting blood glucose observed, indicating the increased insulin might still be below the normal level. Therefore, further investigation focused on identifying administration dose and duration of therapy of exosomes may be necessary. Additionally, a recent study indicated that streptozotocin (STZ)-induced diabetic rats treated with exosomes derived from MSCs display lower blood glucose levels and higher plasma insulin levels, indicating the regeneration of insulin-producing beta-cells (92). Histopathological examination also proved that there is an increase in the size and number of beta-cells with decreasing fibrosis and inflammation of the islets. Moreover, in comparison with their parent cells, MSC-derived exosomes showed superior therapeutic and regenerative results (92). In fact, some researchers have stated that exosomes can be used as an alternative to whole stem cell therapies because they are safer, faster, and easier to inject, with more efficient outcomes and longer storage times than stem cells (92, 111). However, more research may be needed to elucidate the reason why exosomes encompass greater regenerative ability than MSCs themselves, and which substances inside exosomes actually function. In addition to enhancing beta-cell regeneration and function, MSC-derived exosomes also have immunomodulatory effects (112–114). *In vitro* studies demonstrate that EVs derived from bone marrow MSCs induce regulatory dendritic cells and inhibit the proinflammatory responses of T cells against the GAD antigen in patients with T1DM (88, 115). *In vivo* experiments indicated that exosomes derived from adipose tissue-derived MSCs exert protective effects on STZ-induced T1DM mice by increasing the population of regulatory T cells and their products without increasing the proliferation index of lymphocytes (89). All these findings suggest that EVs can mimic the immunoregulatory properties of MSCs and better understanding of involved mechanisms will benefit cell-free therapeutic application.

### Stem Cell-Derived Exosomes and T1DM Complications

Moreover, some animal experiments have indicated that exosomes can also ameliorate diabetic complications (99, 116). Rat bone marrow MSC-derived exosomes can improve cognitive impairment in STZ-induced diabetic mice by repairing damaged neurons and astrocytes, thus reversing dysfunction (98). Although this study shows the exosomes released from MSCs boost impaired neuronal functions, the involved specific proteins or RNA are not identified. Another study showed that exosomal miR-let7c derived from MSCs attenuated kidney injury by preventing renal fibrosis in C57BL/6J mice, which are susceptible to diet-induced obesity and T2DM, with unilateral ureteral obstruction (100). An *in vivo* study indicated that exosomes released by human urine-derived stem cells can prevent podocyte apoptosis and promote cell survival as well as vascular regeneration in rats with T1DM (93). Future studies to clarify the underlying mechanisms and pathways of exosomes on preventing diabetic kidney impairment are necessary. Additionally, exosomes isolated from human endothelial progenitor cells can facilitate cutaneous wound healing by promoting angiogenic activity and vascular endothelial function in diabetic rats and mice (96, 117). Similarly, future research should focus on determining the exact components within exosomes contributing to wound healing of diabetic patients before clinical use. In addition to biomolecule delivery, exosomes can also be applied to deliver synthetic drugs, such as curcumin, that can ameliorate neurovascular dysfunction after stroke in T1DM (43). In conclusion, the animal studies mentioned above indicate that stem cell-derived exosomes have great potential to treat T1DM and diabetic complications and further investigation should elucidate their clinical value in patients with T1DM. However, it should be considered that exosomes could still allow existing tumors to invade the immune system because they can promote cell survival, stimulate angiogenesis, and modulate immunity, although they exhibit a greatly decreased risk of carcinogenesis and maldifferentiation compared with MSCs (118, 119).

### Exosomes and Islets Transplantation

Encouragingly, some research suggests that exosomes might promote the survival of transplanted pancreatic islets and enhance the efficiency of this treatment (120). The cross-talk between endothelial cells and beta-cells is critical for islet transplantation because it is associated with the revascularization process. *In vitro* experiments indicate that human islet-derived exosomes carrying angio-miRNAs can be captured by intraislet endothelial cells and favor angiogenesis and engraftment (85). Further studies should focus on evaluating whether they can be applied in inhibiting ischemia-reperfusion injury in solid organ and cell transplantation. It has also been reported that MVs released from endothelial progenitor cells can activate an angiogenic program and sustain vascularization in SCID (severe combined immunodeficient) mice, which lack both T and B lymphocytes (121). Furthermore, islet-derived exosomes have been observed to induce the expression of proangiogenic and antiapoptotic factors and inhibit antiangiogenic and proapoptotic molecules in islet endothelial cells (85). In addition to promoting revascularization, exosomes can improve islet transplantation through immunomodulatory effects. A study indicated that MSC-derived exosomes can improve islet transplantation by enhancing regulatory T cell function and inhibiting peripheral blood mononuclear cell (PBMC) proliferation (97). For safety concerns, the dose of factors inside exosomes needs to be accurately investigated. In summary, in the context of islet transplantation, exosomes may represent an exciting new therapy not only for the improvement of revascularization but also for the induction of transplant tolerance.

There are several practical problems that should be taken into consideration before any clinical use. First, the cell origin of exosomes affects their distribution, suggesting organotropic characteristics (104, 122). Therefore, modification of the exosome membrane may increase binding to specially targeted cells. Moreover, the route of administration, including intraperitoneal or intramuscular administration, can decrease the accumulation of exosomes in the liver, potentially leading to a higher concentration in target organs, such as the pancreas (122). Finally, the timing and clearance pattern of exosomes should be investigated. Studies show that macrophage-depleted mice display slower disappearance of injected exosomes, suggesting that macrophages may be associated with exosome clearance (123).

## Challenges and Prospects

In the past few decades, exosomes have shown great potential in development of autoimmune diseases, including T1DM. However, their basic and applied research is still in the early stage, and many challenges must be overcome. First, in all studies associated with exosomes, the isolation, purification and identification process is the first and the most important step. Nowadays, the most effective technique to get exosomes is differential ultracentrifugation, which cannot obtain exosomes with 100% purity. Exosomes in most T1DM studies actually represent mixed EV populations, mainly including exosomes and MVs. Therefore, the further research should focus on developing the specific markers to distinguish different subtypes of EVs. In addition, one issue in the application of exosomes for diagnostic markers is process portability, so unified methodologies for the isolation, purification, and characterization of exosomes should be generated before translation to clinical practice. Moreover, exosomes isolated from biofluids such as blood derive from multiple different tissues and organs. However, no clear surface markers have been identified for exosomes from different cell types. In the context of T1DM, the abnormality of both pancreatic islets and immune system contributes to its pathological process and many kinds of cells take part in its onset and development. Thus, developing approaches to determined origination of exosomes will be beneficial to clarify their functions in T1DM and reveal the underlying mechanisms of this disease. Finally, exosomes have shown “double-edged sword” characteristic, not only promoting, but also suppressing diseases progression, such as tumors (124). Therefore, identifying exosomes subgroups on the basis of their functions is equally critical.

## Conclusion

Currently, the treatment of T1DM and its related complications is associated with an enormous economic burden for both society and individuals. Early identification of high-risk individuals do not catch T1DM is critical to implement timely preventive measures and avoid or delay disease exacerbation. Additionally, more comprehensive knowledge of the pathophysiological process will help us treat the root cause of diabetes rather than relieving its symptoms only. For the past few years, EVs, especially exosomes, have emerged as important agents mediating intercellular communication. Exosomes take part in not only physiological processes in the body but also pathological conditions. Accumulated evidence has shown that they are involved in the onset and development of diabetes and that disease conditions alter the number and cargo of exosomes. Therefore, a better understanding of exosomes will help us reveal the underlying pathogenic mechanisms of T1DM, provide novel biomarkers for diagnosis, and lead to the development of new therapeutic strategies.

## Author Contributions

HP searched references, wrote the first draft of the paper, and revised the text. SL, YAX, YIX, XL, and GH critically revised the text and provided substantial scientific contributions. ZX and ZZ proposed the project and revised the manuscript. All authors contributed to the article and approved the submitted version.

## Funding

This work was supported by the National Natural Science Foundation of China (grant numbers 81873634, 81400783, 82070813), the National Key R&D Program of China (grant numbers 2016YFC1305000, 2016YFC1305001, 2018YFC1315603), the Science and Technology Major Project of Hunan Province (grant number 2017SK1020), Hunan Province Natural Science Foundation of China (Grant No. 2018JJ2573, 2020JJ2053).

## Conflict of Interest

The authors declare that the research was conducted in the absence of any commercial or financial relationships that could be construed as a potential conflict of interest.
